# The potential role of omentin-1 in obesity-related metabolic dysfunction-associated steatotic liver disease: evidence from translational studies

**DOI:** 10.1186/s12967-023-04770-8

**Published:** 2023-12-11

**Authors:** Noel Salvoza, Pablo Giraudi, Silvia Gazzin, Deborah Bonazza, Silvia Palmisano, Nicolò de Manzini, Fabrizio Zanconati, Alan Raseni, Francesca Sirianni, Claudio Tiribelli, Natalia Rosso

**Affiliations:** 1 Fondazione Italiana Fegato, ONLUS Area Science Park, Basovizza, Trieste, Italy; 2 School of Molecular Biomedicine, University of Trieste, Trieste, Italy; 3 Surgical Pathology Unit, Cattinara Hospital, ASUGI, Trieste, Italy; 4 Department of Medical, Surgical and Health Sciences, University of Trieste, Trieste, Italy; 5 Clinical Chemistry Urgency Laboratory Spoke, IRCCS Burlo Garofolo Paediatric Hospital, Trieste, Italy

**Keywords:** MASLD, MASH, Obesity, omentin-1, Translational models, VAT

## Abstract

**Background:**

Obesity, characterized by visceral adipose tissue (VAT) expansion, is closely associated with metabolic dysfunction-associated steatotic liver disease (MASLD) and metabolic dysfunction-associated steatohepatitis (MASH). Recent research has highlighted the crucial role of the adipose tissue—liver axis in the development of MASLD. In this study, we investigated the potential role of omentin-1, a novel adipokine expressed by VAT, in obesity-related MASLD pathogenesis.

**Methods:**

Through in silico analysis of differentially expressed genes in VAT from obese patients with and without MASH, we identified omentin-1 as a significant candidate. To validate our findings, we measured omentin-1 levels in VAT and plasma of lean controls and obese patients with biopsy-proven MASLD. Additionally, we assessed omentin-1 expression in the VAT of diet-induced mice MASLD model. In vitro and ex vivo studies were conducted to investigate the effects of omentin-1 on MASLD-related mechanisms, including steatosis, inflammation, endoplasmic reticulum (ER) stress, and oxidative stress. We also analyzed the impact of d-glucose and insulin on VAT omentin-1 levels ex vivo.

**Results:**

Compared to the lean group, the obese groups exhibited significantly lower VAT and plasma levels of omentin-1. Interestingly, within the obese groups, omentin-1 is further decreased in MASH groups, independent of fibrosis. Likewise, VAT of mice fed with high-fat diet, showing histological signs of MASH showed decreased omentin-1 levels as compared to their control diet counterpart. In vitro experiments on fat-laden human hepatocytes revealed that omentin-1 did not affect steatosis but significantly reduced TNF-α levels, ER stress, and oxidative stress. Similar results were obtained using ex vivo VAT explants from obese patients upon omentin-1 supplementation. Furthermore, omentin-1 decreased the mRNA expression of *NF-κB* and mitogen-activated protein kinases (*ERK* and *JNK*). Ex vivo VAT explants showed that d-glucose and insulin significantly reduced omentin-1 mRNA expression and protein levels.

**Conclusions:**

Collectively, our findings suggest that reduced omentin-1 levels contribute to the development of MASLD. Omentin-1 supplementation likely exerts its beneficial effects through the inhibition of the NF-κB and MAPK signaling pathways, and it may additionally play a role in the regulation of glucose and insulin metabolism. Further research is warranted to explore omentin-1 as a potential therapeutic target and/or biomarker for MASLD.

**Graphical Abstract:**

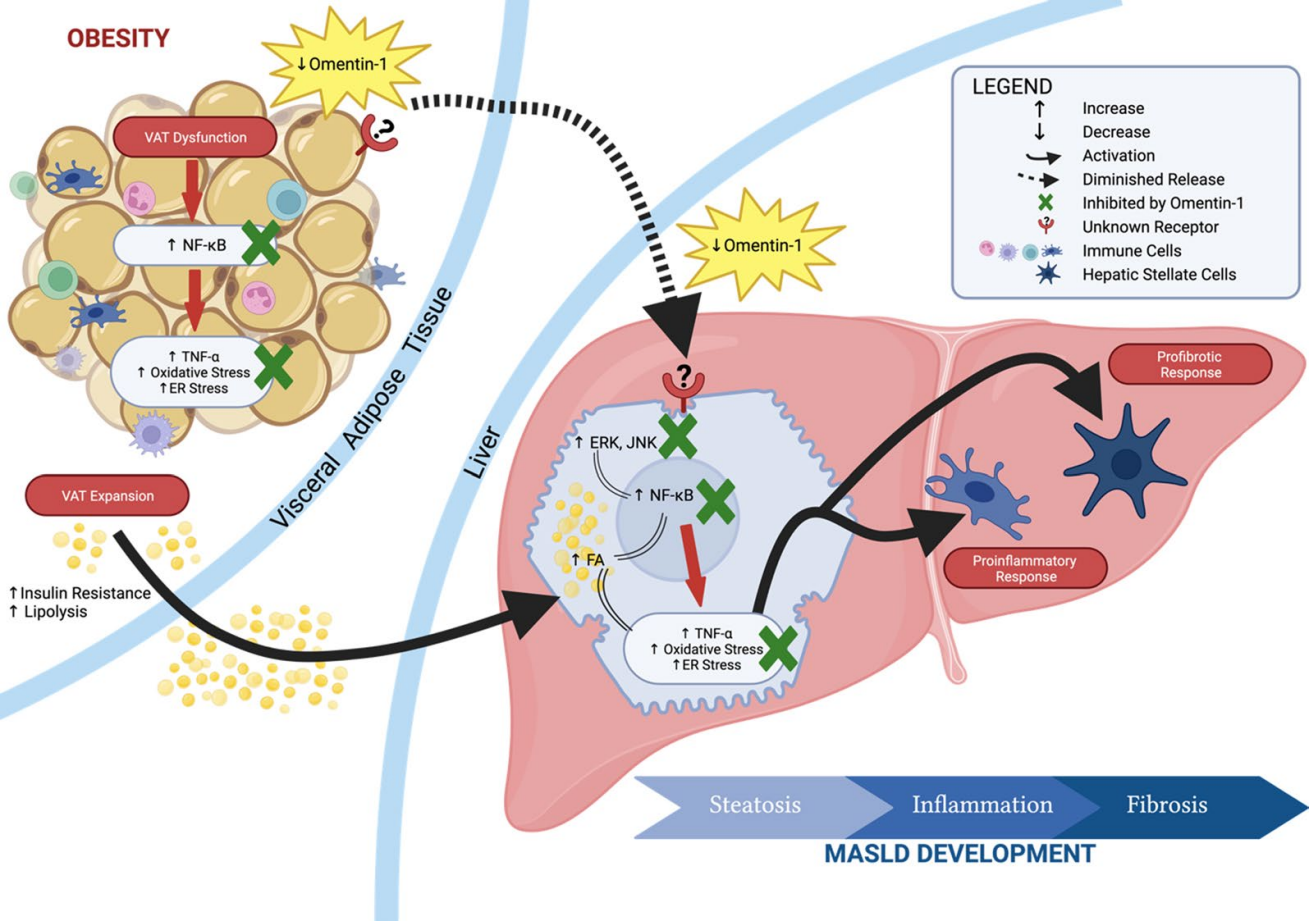

**Supplementary Information:**

The online version contains supplementary material available at 10.1186/s12967-023-04770-8.

## Introduction

Metabolic dysfunction-associated steatotic liver disease (MASLD), formerly known as nonalcoholic fatty liver disease (NAFLD) is a serious public health issue, with an estimated global prevalence of 25.24% [1]. The prevalence of metabolic dysfunction-associated steatohepatitis (MASH), a more severe form of MASLD, is estimated to be around 7.6% [2]. MASH is associated with liver-related related outcomes such as cirrhosis, liver failure and hepatocellular carcinoma, while non-liver-related adverse outcomes are mostly linked to cardiovascular disease and malignancy [1, 3].

Most of the epidemiological studies reported that the prevalence of MASLD is higher in individuals with metabolic risk factors such as obesity, type 2 diabetes mellitus (T2DM), dyslipidemia, and metabolic syndrome (MS). The presence of obesity alone, in the absence of other features of MS, increases the risk of developing MASLD. Obesity, characterized by adipose tissue (AT) mass expansion, is seen in 51% of MASLD and 81% of MASH patients globally [1]. Fat accumulation in the organs, especially the visceral tissue, leads to their dysfunction, promoting ectopic fat accumulation in the liver, inflammation, endoplasmic reticulum (ER) stress, oxidative stress, and impairment of glucose metabolism, among others [4]. Aside from its role as the regulator of lipid flux to the liver, AT is also recognized as a major endocrine organ producing a large array of mediators, known as adipocytokines [5]. The role of adipocytokines in AT-liver crosstalk has become an important area of MASLD research because of the potential utility of those proteins/mediators, as diagnostic markers and/or therapeutic targets—since no reliable diagnostic marker and pharmacological treatment are currently approved for the disease.

Omentin-1 (also known as intelectin-1), a novel adipocytokine, is a peptide of 313 amino acids containing a secretory signal sequence and a fibrinogen-related domain [6]. Omentin-2, a homolog with 83% amino acid identity with omentin-1 is found in the same chromosomal region [7]. Several reports indicated that omentin-1 and -2 are highly expressed in visceral adipose tissue (VAT), but omentin-1 was shown to be the major circulating isoform in human plasma [8]. Omentin-1 was observed to be secreted exclusively into the culture medium of VAT, not subcutaneous adipose tissue (SAT), with stromal vascular cells playing a primary role in its production over adipocytes within VAT [6].

Plasma omentin-1 level decreases in overweight and obese humans, while it increases after obese patients lose weight or after taking antidiabetic drugs [9, 10]. Regarding its biological activity, omentin-1 enhances insulin-stimulated glucose uptake via Akt (protein kinase B) activation in human adipocytes, suggesting its role in T2DM susceptibility [6]. Further, omentin-1 exerts anti-inflammatory effect by ameliorating macrophage activation via inhibiting the NF-κB pathway in obese mice [11]. Both insulin resistance and inflammation are associated with MASLD. These two molecular mechanisms, along with steatosis, oxidative stress, ER stress, and fibrosis, are key pathologic drivers in MASLD development.

In this study, we conducted a simple in silico analysis identifying omentin-1 and investigated its role in MASLD for the first time, using different translational approaches. We simultaneously determined the expression of omentin-1 in VAT at both mRNA and protein levels in MASLD patients and mice fed with high-fat diet (HFD). Furthermore, we present novel data regarding the plasma levels of omentin-1 in obese subjects with different stages of MASLD. Successively, to elucidate its role in the liver and VAT, we evaluated its beneficial effects in the MASLD-related pathophysiological mechanisms such as steatosis, inflammation, ER stress, and oxidative stress.

## Materials and methods

### In silico strategy

The literature review followed the Preferred Reporting Items for Systematic Reviews and Meta-Analyses (PRISMA) guidelines, with the paper by du Plessis et al. [12]. being chosen for its similarity to our morbidly obese cohort and the availability of VAT datasets. The gene expression data set GSE58979 was downloaded from Gene Expression Omnibus (GEO), which included 9 obese VAT samples (group 1) and 7 MASH VAT samples (group 3). Differentially expressed genes (DEGs) were identified using GEO2R through limma method (see Additional file 1). The significance of DEGs was calculated by the t-test and was represented by the p-value. The threshold for the DEGs was set as corrected p-value < 0.05 and log_2_ fold change (FC) of |1|.

In our systematic strategy, the identifiers (IDs) for protein-coding genes in the consulted data resources were standardized, through mapping to the UniProtKB identifiers on UniProt database, and only those IDs were further used. Moreover, datasets of our interest collected from Human Protein Atlas (HPA) were used as in silico sieve filters. Dataset comparison and sub-groups selection was performed by applying Venn diagrams using InteractiVenn web-based tool. Venn diagrams were used as in silico filters to identify the interested proteins, those fulfilling the following desired criteria: visceral adipose tissue-enriched, secreted proteins, secreted in plasma or blood, and not included as part of the housekeeping proteome. We finally selected omentin-1 as the most pertinent gene for our subsequent analysis as it fulfils all the criteria, and it is the only adipocytokine on the list (see Additional file 2).

### Study participants

The assessment of omentin-1 VAT expression and plasma level was performed retrospectively in a morbidly obese (MO) cohort enrolled in a bariatric surgery program. All patients gave their written consent, and the study has been approved by the local Ethical Committee under protocol N. 22979 (Comitato Etico Regionale Unico, FVG, SSN, Italy). The MO cohort was stratified according to obese (Ob) group = 19; obese MASH (Ob-M) group = 20; and obese MASH with fibrosis (Ob-MF) group = 16. The baseline characteristics of the MO cohort are shown in Additional file 3. In addition, a total of 17 lean controls with BMI of 18.5–24.9 kg/m^2^ were included in the ELISA study. For PCR and western blot, VAT from 5 lean study participants were used as controls.

### In vitro model of hepatic steatosis

Hepatoma cell line Huh7 (JHSRRB, Cat #JCRB0403) was obtained from the Health Science Research Resources Bank (Osaka, Japan) and grown in DMEM-HG with 10% FBS. Huh7 cells were exposed for 24 h to 1200 µM of free fatty acids (FFA) (oleic:palmitic ratio 2:1 µmoL/µmoL) as previously described by our group [13]. To determine the experimental concentration, the cytotoxic effect of FFA (1200 µM) and recombinant omentin-1 (Bio Vendor, Candler, NC, USA), alone or in combination, was assessed by MTT colorimetric assay after 24 h.

### Ex vivo primary explant culture of VAT

VAT explants from morbidly obese MASH patients (without T2DM) undergoing bariatric surgery were cultured using the modified protocols of Carswell et al. [14] and Tan et al. [15]. Within 30 min after the surgery, tissue was minced into small pieces, approximately 5–10 mg per piece (∼ 1–2 mm^3^) and transferred into six-well plates (∼100 mg/well) containing 3 mL of appropriate medium. VAT explants were cultured for 24 h with or without the addition of insulin (10^−5^ M, 10^−7^ M) or d-glucose (50 mmol/L, 25 mmol/L).

### Animal model (in vivo)

C57Bl/6 mice pups were provided by local specific pathogen-free (SPF) animal facility (University of Trieste). Immediately after weaning, mice were housed (22 °C ± 2 °C) in a 12 h light/dark schedule, and fed ad-libitum with control diet (CD, 811900 Special Diets Services, England) or HFD diet (D12331, Research Diets, New Brunswick, NJ, USA) plus 42 g/L fructose/sucrose in drinking water, as previously described [16]. Based on the knowledge of the model and the experimental goals, diet was continued for 3 weeks and 20 weeks. Liver and epididymal fat (a depot of VAT) were dissected for the histologic evaluation and experimental use, respectively. Blood tests and histology were performed as previously described [16]. All experimental protocols were approved by the local OPBA (Organismo Per il Benessere dell’Animale) and by the Competent National Authority (Ministero della Salute-Direzione Generale della Sanità Animale e dei Farmaci Veterinary Approval 56/2022PR).

### Fluorometric determination of intracellular fat content

Intracellular fat content in vitro was determined by flow cytometry using Nile red staining, a vital lipophilic dye used to label fat accumulation in the cytosol. After 24 h of FFA exposure (with or without omentin-1 treatment), intracellular fluorescence was detected using a Becton Dickinson FACSCalibur System on the FL2 emission channel through a 585 ± 21 nm bandpass filter, following excitation with an argon-ion laser source at 488 nm. Data were collected in 10,000 cells and analyzed using FlowJo (Tree Star Inc., Ashland, OR, USA) analysis software.

### Quantitative PCR

Total RNA was extracted from cell culture harvest and homogenized VAT using Tri-reagent kit (Sigma-Aldrich, MO, USA). cDNA was generated with High Capacity cDNA Reverse Transcription Kit (Applied Biosystems, Waltham, MA, USA). Quantitative PCR was performed in CFX Connect Real-Time PCR Detection System (Bio-Rad, Hercules, CA USA) in a specific reaction volume containing 25 ng of cDNA, 1X iQ SYBR Green Supermix, and primer pairs. The relative quantification was made using the Pfaffl modification of the ΔΔCt equation, considering the efficiencies of individual genes and housekeeping genes.

### Western blot analysis

The following primary antibodies were used: Omentin-1/Intelectin-1 1:100 (Santa Cruz Biotech, Santa Cruz, CA, USA) and the reference α-tubulin 1:2000 (Santa Cruz Biotech, Santa Cruz, CA, USA). Blots were incubated with anti-mouse IgG-HRP-conjugated secondary antibody (1:500 Omentin-1/Intelectin-1 and 1:2000 for α-tubulin). Protein bands were visualized using the ECL immunoblotting detection system (GE Healthcare, Buckinghamshire, UK) and developed on a C-DiGit® Blot Scanner (LI-COR Biosciences, NE, USA). Results were expressed as the ratio of omentin-1 protein expression to that of a reference protein, α-tubulin. Relative densitometry analyses of the immunoblots were determined using IMAGE STUDIO software.

### Glutathione content assay Superoxide dismutase (SOD) activity assay

The simultaneous assay for both GSH (reduced) and GSSG (oxidized) was done using the modified protocol of Mokrasch and Teschke [17] and were normalized to total µg of proteins. Total SOD activity was measured using a commercial kit (Sigma-Aldrich, MO, USA).

### Omentin-1 and TNF-α ELISA

The plasma level of omentin-1 in patients was measured using Human Omentin-1 ELISA Kit (BioVendor, RD191100200R) and the TNF-α levels of Huh7 and VAT supernatants were quantified by Human TNF alpha ELISA Kit (BioVendor, RAF128R). The levels of protein analytes were normalized to total µg of proteins.

### Statistical analysis

Unless indicated otherwise, all values are presented as mean ± standard deviation (SD). The normal distribution of variables was evaluated by Kolmogorov–Smirnov test. Differences between two groups were assessed using the Mann–Whitney U test or student’s t-test. Data involving more than two groups were assessed by One-way-ANOVA or Kruskal–Wallis test, followed by post-hoc analysis. Spearman rank correlation was used for the calculation of associations between variables. Specific analysis details are indicated in figure legends. Statistical significance was determined at p < 0.05. All figures and statistical analyses were generated using GraphPad Prism 9 and SPSS 29, respectively.

## Results

### In vivo clinical validation

**Fig. 1:**
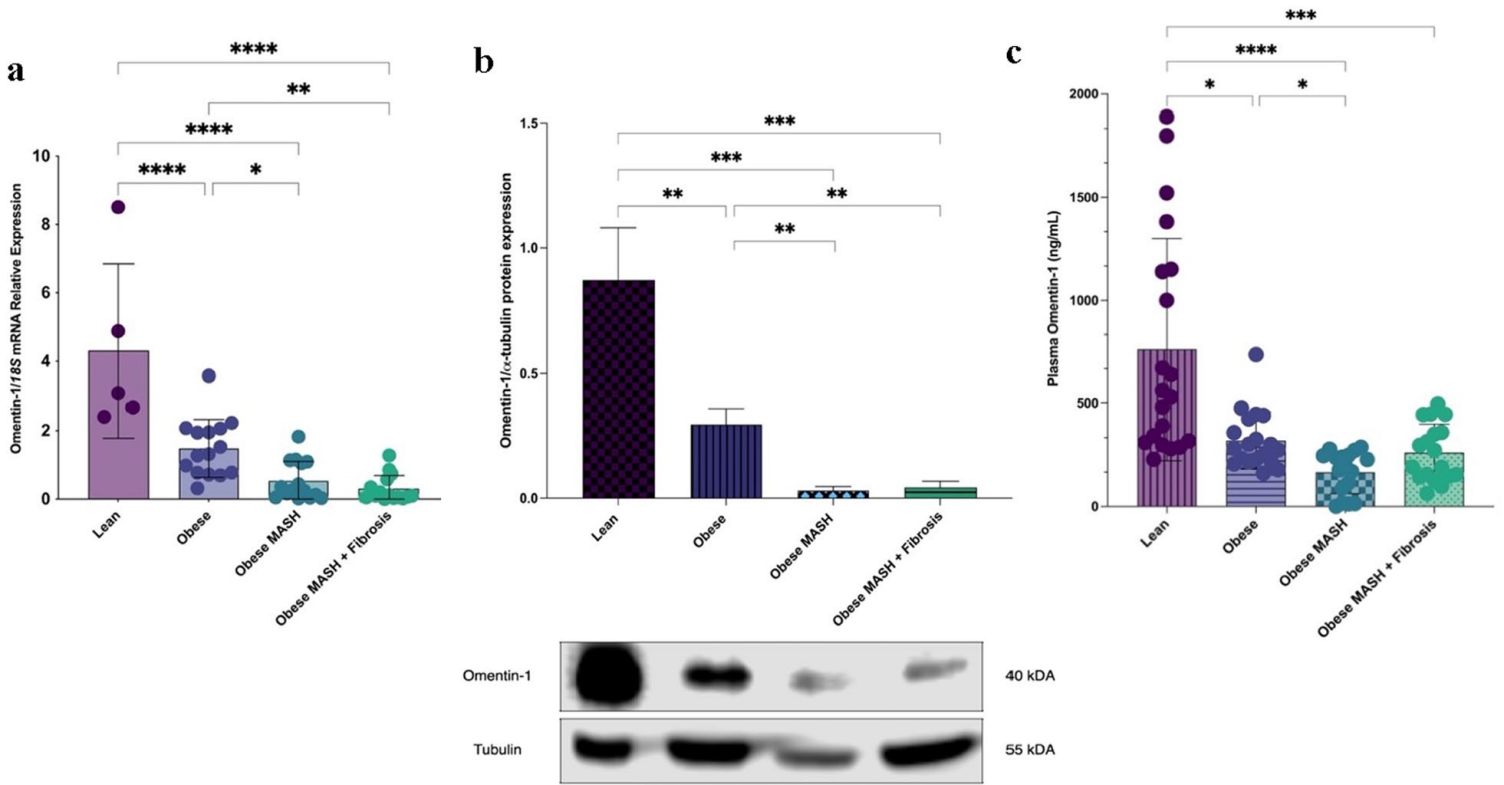
Human VAT omentin-1 **a** mRNA expression, **b** protein expression, and **c** plasma levels in obese groups and lean controls. Omentin-1 mRNA expression is significantly decreased in the VAT of all obese groups as compared to the lean controls (N = 60). Representative blot and densitometric analysis of omentin-1 normalized to α-tubulin revealed that protein expression is also significantly decreased in the VAT of all obese groups as compared to the lean controls (n = 3/group). For plasma levels, values presented are the mean ± SD of individual patients (N = 72). Group comparison by Kruskal–Wallis and post hoc Dunn’s test. *p < 0.05, **p < 0.01, ***p < 0.001, ****p < 0.0001

Based on in silico analysis, omentin-1 is one of the downregulated genes in VAT of obese MASH. In vivo clinical validation showed decreased omentin-1 mRNA expression in all obese groups as compared to lean controls, independent of the presence of fibrosis (Fig. 1a). Further, the changes noted at the mRNA level were also reflected at the protein level (Fig. 1b). Likewise, plasma omentin-1 levels were lower in the obese groups than in the lean control group (Fig. 1c).

Having shown that the plasma level of omentin-1 in obese groups differ, we investigated the relationship of omentin-1 with the clinical and biochemical parameters (Table 1). Omentin-1 plasma level had a significant positive correlation with omentin-1 mRNA (ρ = 0.382 *p* = 0.013) and a significant negative correlation with total cholesterol (ρ = − 0.307, *p* = 0.022). Interestingly, omentin-1 plasma level negatively correlates with ALT (ρ = − 0.279, *p* = 0.039) but not AST. Further, the AST/ALT ratio positively correlates with the omentin-1 plasma level (ρ = 0.285, *p* = 0.042).

**Table 1:** Correlation of clinical and laboratory parameters with plasma omentin-1 level

Variable	rho	*p* value
Omentin-1 mRNA	0.382	0.013*
BMI (kg/m^2^)	− 0.230	0.093
Fasting glucose (mg/dL)	0.026	0.851
AST (UI/L)	− 0.206	0.136
ALT (UI/L)	− 0.279	0.039*
AST/ALT ratio	0.285	0.042*
GGT	− 0.049	0.723
ALP (U/L)	− 0.034	0.81
Triglycerides (mg/dL)	− 0.212	0.119
Total cholesterol (mg/dL)	− 0.307	0.022*
HDL (mg/dL)	− 0.029	0.836
Insulin (µU/mL)	− 0.159	0.333
Platelet (×10^9^ L)	− 0.076	0.581

Pearson’s or Spearman’s correlation coefficient (Rho) measures the strength and direction of association between the two variables under study (N = 55)

*p < 0.05

### VAT omentin-1 expression in HFD mice

Human omentin-1 gene is 80–85% homologous to mice omentin-1 [18]. To investigate the mouse omentin-1 level during diet-induced obesity, we assigned C57Bl/6 littermates to receive either control or high-fat diet, supplemented with fructose/sucrose in drinking water, as previously described by our group [16].

**Fig. 2:**
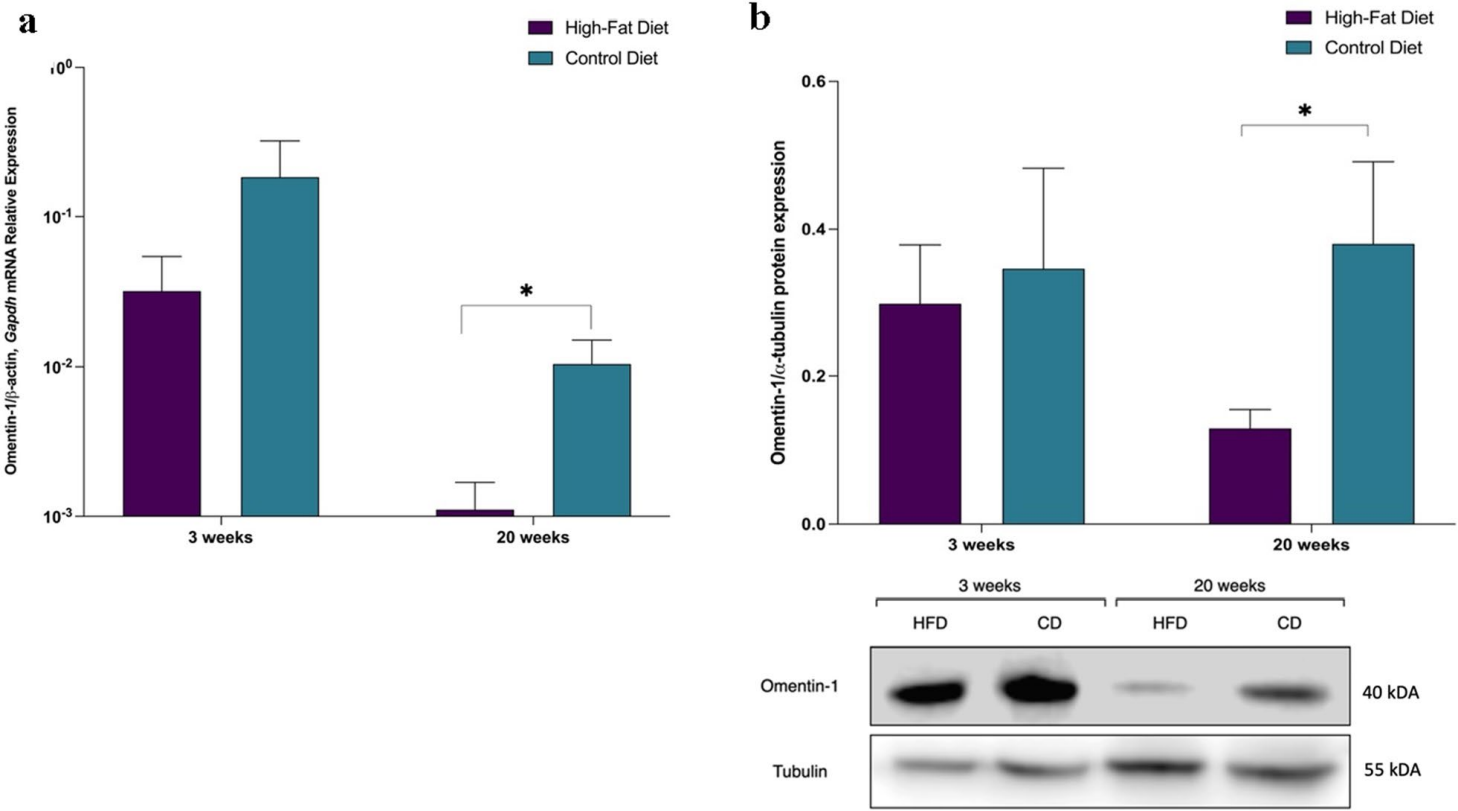
VAT omentin-1 **a** mRNA expression and **b** protein expression HFD mice and control diet mice. 20 weeks mice fed with HFD (n = 18), showing histological signs of MASH, have significantly lower expression compared to mice fed with a control diet (n = 13). Representative blot and densitometric analysis of omentin-1 normalized to α-tubulin revealed that protein expression is significantly decreased in the VAT of HFD mice as compared to control mice at 20 weeks (n = 3–5 mice/group). *p  < 0.05

Mice treated with HFD for 20 weeks developed obesity, dyslipidemia, hyperglycemia, hyperinsulinemia, insulin resistance, and histological signs of MASH as compared to control diet mice (Table 2). Real-time PCR showed decreased mRNA expression of omentin-1 in the VAT of HFD versus control diet mice at 20 weeks (Fig. 2a). Similarly, Western blot analysis from representative mice VAT also confirmed the decreased omentin-1 expression in HFD mice versus control (Fig. 2b).

**Table 2:** Anthropometric, biochemical, and histological characteristics of mice

Variable	3 weeks		*p* value	20 weeks		*p* value
	HFD (n = 8)	CD (n = 7)		HFD (n = 18)	CD (n = 13)	
Sex (female)	4 (50%)	4 (57.1%)	0.782	11 (61.1%)	7 (53.8%)	0.686
Body weight (g)	21.3 ± 2.61	18.9 ± 2.25	0.083	39.50 ± 7.38	28.06 ± 3.79	< 0.001***
Body length (cm)	8.65 ± 0.35	8.36 ± 0.42	0.160	9.27 ± 0.30	8.87 ± 0.32	< 0.001***
BMI (kg/m^2^)	28.38 ± 2.33	27.14 ± 2.61	0.351	45.82 ± 7.11	35.69 ± 3.40	< 0.001***
Total cholesterol (mg/dL)	141.50 ± 17.53	86.14 ± 10.75	< 0.001***	168.22 ± 43.45	85.69 ± 11.71	< 0.001***
HDL (mg/dL)	98.14 ± 13.93	59.43 ± 6.05	< 0.001***	111.78 ± 25.19	63.69 ± 11.88	< 0.001***
LDL (mg/dL)	82.03 ± 14.42	33.84 ± 9.48	< 0.001***	110.02 ± 36.82	36.61 ± 10.05	< 0.001***
Triglycerides (mg/dL)	110.00 ± 34.65	87.14 ± 24.31	0.089	74.33 ± 22.47	69.54 ± 15.93	0.516
AST (U/L)	83.60 ± 40.15	73.60 ± 28.04	0.660	247.44 ± 181.57	69.44 ± 20.40	0.008**
ALT (U/L)	57.18 ± 33.68	40.20 ± 29.61	0.092	67.35 ± 31.64	46.08 ± 35.41	0.047*
Glucose (mg/dL)	329.25 ± 26.00	307.71 ± 34.04	0.188	321.94 ± 79.11	271.00 ± 35.00	0.039*
Insulin (µU/mL)	1.33 ± 0.71	0.96 ± 0.25	0.219	2.79 ± 1.88	1.22 ± 0.30	0.042*
HOMA-IR	1.08 ± 0.57	0.73 ± 0.22	0.162	2.38 ± 1.78	0.87 ± 0.28	0.010**
Steatosis grade (0/1/2/3)	75%/25%/ 0/0	100%/0/ 0/0	0.155	5.6%/66.7%/ 16.7%/11.1%	100%/0/0/0	< 0.001***
Lobular inflammation (0/1/2/3)	62.5%/25%/ 12.5%/0	71.4%/28.6%/ 0/0	0.626	27.8%/27.8%/ 33.3%/11.1%	76.9%/23.1%/ 0/0	0.022*
Ballooning (no/yes)	100%/0	100%/0	–	93.3%/6.7%	100%/0	–

Data are shown as mean ± SD for continuous variables, number (%) for binary variables, and frequency for categorical variables. T-test was used to test for significant differences with continuous variables. Chi-Square test was used for categorical variables

***p < 0.001, **p < 0.01, *p < 0.05

### In vitro effects of omentin-1 on fat-laden hepatocytes

**Fig. 3:**
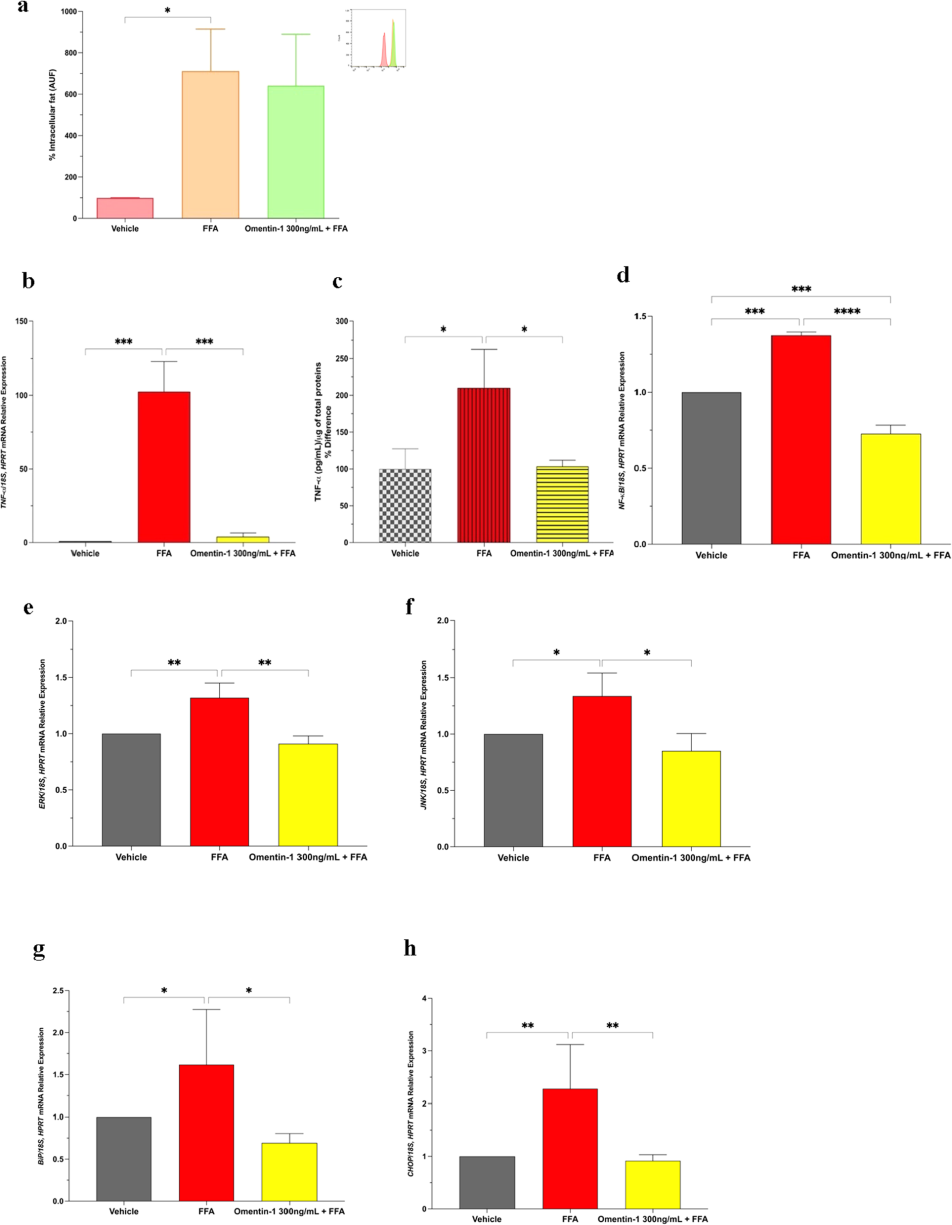

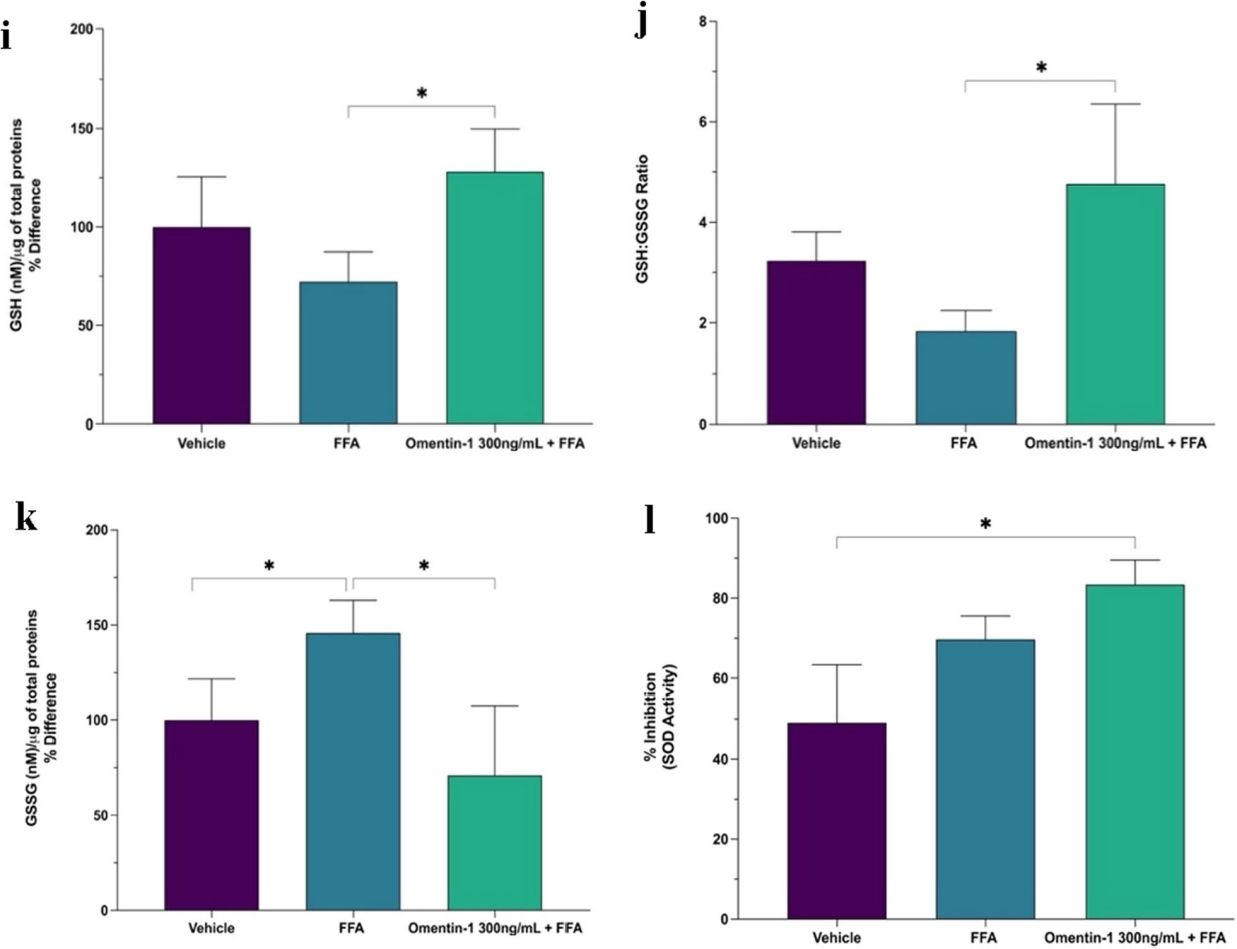
**a** Effect of omentin-1 on hepatocyte fat accumulation. **b**, **c** Effect of omentin-1 and FFA co-treatment on the mRNA expression and supernatant release of TNF-α in Huh7 cells with FFA. **d**–**f** Effect of omentin-1 treatment on the *NF-κB, ERK*, and *JNK* mRNA expression in Huh7 cells with FFA. **g**, **h** Effect of omentin-1 treatment on ER stress markers *BiP* and *CHOP* in Huh7 cells with FFA. **i**–**l** Effects of omentin-1 treatment on the production of ROS in Huh7 with FFA. Values presented are the mean ± SD of at least three biological replicates. GSH and GSSG contents were normalized by the total proteins present in the cell lysates (µg) assessed using BCA. *p < 0.05, **p < 0.01, ***p < 0.001, ****p < 0.0001

Nile red staining through flow cytometry revealed that omentin-1 does not affect steatosis (Fig. 3a). Interestingly, co-treatment of FFA with omentin-1 decreased the mRNA expression of *TNF-α* relative to the vehicle control (Fig. 3b). Consistent with the gene expression results, omentin-1 significantly reduced the release of TNF-α in the cell culture supernatant (Fig. 3c).

The mitogen-activated protein kinase (MAPK) and nuclear factor kappa (NF-κB) signaling pathways are known to be important in regulating the expression of proinflammatory cytokines, including TNF-α. We determined the gene expression of *NF-κB p65* in our in vitro model of steatosis, along with the MAP kinases, extracellular signal regulated kinase (*ERK*) and c-Jun N-terminal protein kinase (*JNK*). When co-treated with omentin-1 in fat-laden hepatocytes, there was a significant reduction in *NF-κB* expression, as well as in the expression of both *ERK* and *JNK* (Fig. 3d, f).

To explore the involvement of omentin-1 in hepatocyte ER stress induced by fat overload, we analyzed the expression levels of two ER stress markers (*BiP* and *CHOP* gene markers). The mRNA expression levels of both markers showed a significant increase upon treatment of FFA (Fig. 3g). Moreover, both markers showed a significant decrease upon co-treatment with omentin-1 (Fig. 3h).

The antioxidant role of omentin-1 was evaluated on fat-laden Huh7 cells using glutathione content assay. The reduced glutathione (GSH) and GSH:GSSG ratio showed reduced levels upon FFA treatment and a significant increase upon co-treatment with omentin-1 (Fig. 3i, j). The exposure to FFA significantly increased the oxidized glutathione (GSSG) content vs. the vehicle-treated control (Fig. 3k), indicating a more consistent oxidative stress state. Another important antioxidant defense system is the superoxide dismutase (SOD) enzyme activity. FFA treatment showed increased SOD total activity, probably a compensatory mechanism from oxidative stress. Interestingly, the SOD activity is further enhanced upon co-treatment with omentin-1 (Fig. 3l).

### Ex-vivo effects of omentin-1 on VAT explants

**Fig. 4:**
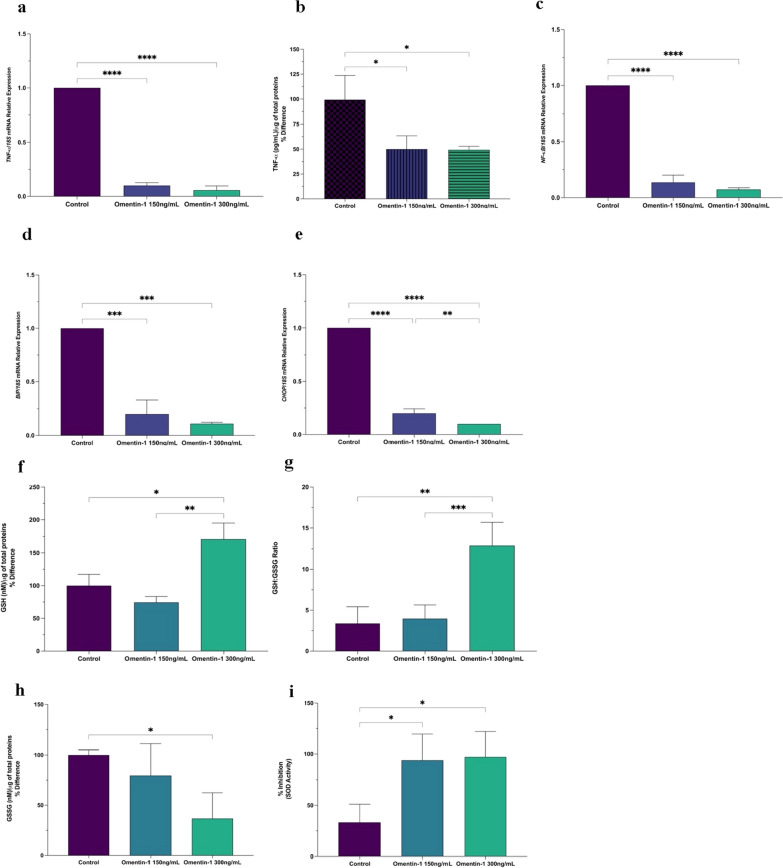
**a**, **b** Effect of omentin-1 and FFA co-treatment on the mRNA expression and supernatant release of TNF-α in VAT explants. **c** Effect of omentin-1 treatment on the *NF-κB* mRNA expression in VAT explants. **d**, **e** Effect of omentin-1 treatment on ER stress markers *BiP* and *CHOP* in VAT explants. **f**–**i** Effects of omentin-1 treatment on the production of ROS in VAT explants. Values presented are the mean ± SD of three biological replicates. GSH and GSSG contents were normalized by the total proteins present in the cell lysates (µg) assessed using BCA. Values presented are the mean ± SD of three patients. *p < 0.05, **p < 0.01, ***p < 0.001, ****p < 0.0001

The addition of recombinant omentin-1 in VAT explants of obese patients significantly reduced the basal *TNF-α* mRNA expression (Fig. 4a) and release in the supernatant (Fig. 4b). Moreover, VAT treated with omentin-1 showed a significant decrease in *NF-κB* mRNA expression versus control (Fig. 4c). The mRNA expression level of *BiP* is significantly reduced upon treatment of omentin-1 (Fig. 4d) while *CHOP* mRNA expression is dose-dependently reduced by omentin-1 as compared to the control (Fig. 4e). The reduced glutathione (GSH) and GSH:GSSG ratio significantly increases upon the addition of omentin-1 300 ng/mL but not omentin-1 150 ng/mL (Fig. 4f, g). On the other hand, the oxidized glutathione (GSSG) almost dose-dependently decreased upon supplementation of omentin-1 (Fig. 4i). Lastly, omentin-1 supplementation significantly enhanced the SOD activity in both concentrations (Fig. 4j).

### Ex vivo effects of d-glucose and insulin on omentin-1 levels

Studies revealed that omentin-1 enhances insulin-stimulated glucose uptake in vitro in both omental and subcutaneous adipocytes and its serum levels are reduced in patients with T2DM and glucose intolerance [6, 10]. Hence, we hypothesized that omentin-1 level might be affected by glucose and insulin modulation.

**Fig. 5:**
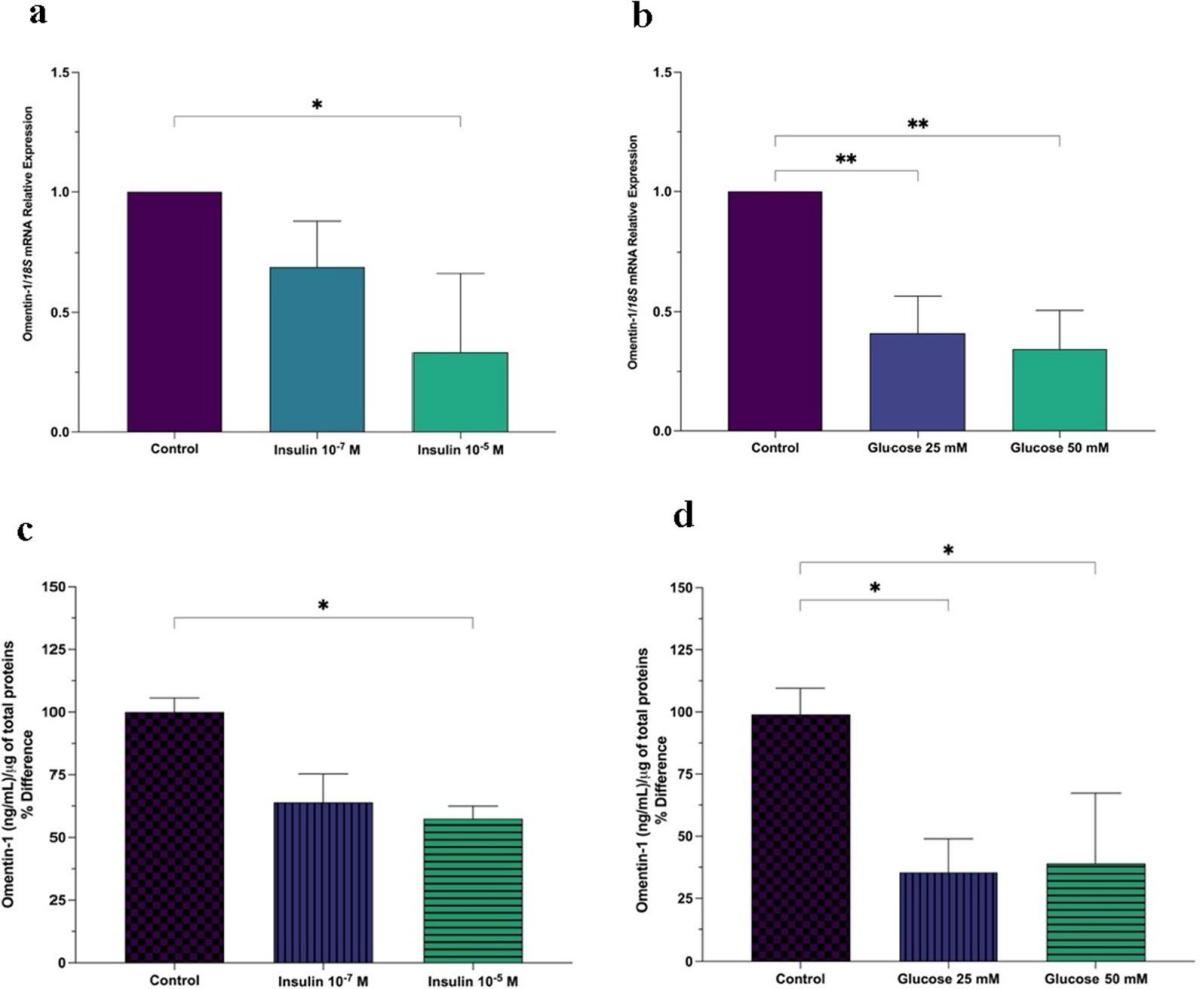
Effects of **a** insulin and **b** glucose on the mRNA expression of omentin-1 in VAT of obese patients; effects of **c** insulin and **d** glucose on the protein level of omentin-1 in VAT of obese patients. Omentin-1 protein level was normalized by the total proteins present in the tissue homogenates (µg) assessed using BCA. Values presented are the mean ± SD of 3–4 patients. *p < 0.05, **p < 0.01

Using the VAT explants from obese MASH patients (without T2DM), we added either insulin or glucose in the medium and determined the levels of omentin-1. Both glucose and insulin resulted in a significant and almost dose-dependent decrease in omentin-1 mRNA expression levels (Fig. 5a, b). Likewise, omentin-1 protein levels were also reduced in VAT homogenates showing consistent results with that of mRNA expression (Fig. 5c, d).

## Discussion

The current study utilized a simple in silico analysis to identify omentin-1 and provided insights regarding its role in MASLD, using a variety of translational approaches. Consistent with previous studies [6, 15], we detected omentin-1 mRNA in VAT but not SAT. Both mRNA and protein levels of omentin-1 in VAT are lower in all obese groups than in lean controls. The expression is further decreased in obese groups with MASH (with or without fibrosis) vs. the Ob group. Additionally, our in vivo mice results concur with our human validation results.

Omentin-1 is reported to be the major circulating form of omentin in human plasma [8]. Interestingly, the results of plasma omentin-1 levels as measured by ELISA are consistent with our VAT mRNA and protein data. Successively, correlation analyses were performed to evaluate the relationship between biochemical parameters and plasma omentin-1 levels in morbidly obese patients. In our study, omentin-1 plasma levels were found to be positively correlated with AST/ALT ratio and negatively correlated with ALT level. These results indicate an association between liver damage and omentin-1 secreted by VAT, supporting the crosstalk theory between the two organs.

Our in vivo findings suggest that a reduced level of omentin-1 is associated with MASLD development, probably via VAT-liver crosstalk. Furthermore, when considering only the obese groups in our study, it is unlikely that BMI is solely responsible for their lower omentin-1 levels since all patients had a BMI of > 35 kg/m^2^. Therefore, we also hypothesized that the further decrease in omentin-1 level could be an additive effect of MASLD severity to obesity. As such, the severity of MASLD results from several pathophysiological mechanisms, such as oxidative stress and ER stress, inflammation, and glucose-insulin impairment. To answer our hypotheses, we employed in vitro and ex vivo studies to evaluate the role of omentin-1 in MASLD-related pathophysiological mechanisms.

MASLD pathogenesis is complex but the onset of the disease is still represented by the accumulation of fat in the liver [19, 20]. Our group previously developed an in vitro model of MASH where the exposure of hepatocytes to high concentrations of FFA promotes steatosis, inflammation, oxidative stress, and fibrogenic response, similar to those observed in patients with MASLD [21]. Using this in vitro model to represent the pathologic events in the liver, we evaluated the beneficial effects of recombinant omentin-1. In parallel, we also studied its effects in ex vivo VAT explants obtained from obese patients with MASLD to determine its role in the actual diseased tissue setting.

The expression of *TNF-α* in steatotic hepatocytes and VAT supplemented with omentin-1 has not been examined to date. Here we have demonstrated that omentin-1 reduced the levels of *TNF-α* in both fat-laden hepatocytes and VAT explants from obese patients. *TNF-α* is a key mediator in the process of MASLD development by not only promoting inflammatory response, but also mediating insulin resistance, and inducing fibrosis-associated proteins [22]. Moreover, substantial evidence has highlighted the beneficial effects of omentin-1 in other inflammatory-associated disorders such as osteoporosis and inflammatory bowel diseases [23–25]. It has been demonstrated that omentin-1 plays an anti-inflammatory role by preventing the TNF-α-induced COX-2 expression in vascular endothelial cells by inhibiting the AMPK/eNOS/NO pathways [26]. Another study showed that omentin-1 inhibits TNF-α-induced expression of adhesion molecules in endothelial cells by blocking ERK/NF-κB pathway. Therefore, *NF-κB* transcription factor regulates a cascade of inflammatory responses by TNF-α activation [27, 28]. Based on this premise, we also investigated the role of omentin-1 in *NF-κB* expression. Indeed, we found that omentin-1 decreased the expression of *NF-κB* in both fat-laden hepatocytes and VAT explants, suggesting that the anti-inflammatory effect may act via inhibition of this pathway. To further elucidate the role of omentin-1 on *NF-κB* pathway, we investigated its effect on its upstream regulator, MAP kinases. The MAPK family of proteins, including JNK and ERK, exert an influence on NF-κB activation, consequently impacting TNF-α and the inflammatory response [29]. Interestingly, we confirmed by real-time PCR that omentin-1 significantly inhibited the FFA-induced upregulation of ERK and JNK expression.

Obesity, a state of low-grade systemic inflammation, is associated with ROS overproduction and oxidative stress due to mitochondrial dysfunction [30]. As a result, inflammation and oxidative stress are involved in the induction of ER stress signaling pathways and subsequent unfolded protein response (UPR) activation to restore ER homeostasis [31]. This implies that oxidative stress, ER stress, and inflammatory pathways somewhat converge at different stages of obesity resulting in disease progression. Our group and other authors previously reported that fat-laden hepatocytes increase ROS production and ER stress [21, 32, 33]. Similarly, growing evidence suggests that excess energy substrate input associated with obesity enhanced ROS generation and ER stress by VAT [34–36]. Interestingly, we presented herein that adding omentin-1 mitigated both the oxidative stress and ER stress in our in vitro and ex vivo setups. Specifically, a significant decrease in oxidized glutathione (GSSG) levels and enhanced SOD enzyme activities were observed in fat-laden hepatocytes and VAT explants. In line with these observations, our results also showed that ER stress markers (*BiP* and *CHOP*) were also reduced by omentin-1 supplementation. Like omentin-1, vaspin, an AT-secreted substance that has insulin-sensitizing properties, also exerts the same beneficial effects on ER stress–induced metabolic dysfunctions. However, unlike omentin-1, with no known receptor to date, vaspin binds to *BiP*, which is recruited from ER to the plasma membrane under ER stress [37]. Further investigations are needed to elucidate the role of omentin-1 in oxidative stress and ER stress, as well as to identify its specific receptor. Nonetheless, our findings suggest that oxidative stress and ER stress, as well as inflammation, all of which increase in parallel with metabolic dysfunctions, could be alleviated by omentin-1.

Metabolic disorders like obesity, diabetes, and polycystic ovarian syndrome are all characterized by insulin resistance and impairment of glucose metabolism. It has been shown that in vitro supplementation of recombinant omentin-1 enhances insulin-mediated glucose uptake by adipocytes via GLUT4 translocation and Akt phosphorylation [6, 15]. Furthermore, as reported herein, omentin-1 decreases the ER stress marker *BiP*, which is also thought to maintain glucose uptake in glucose storage tissues [37]. Thus, given the role of omentin-1 in glucose homeostasis, we hypothesized that hyperinsulinemia and hyperglycemia decrease its expression. We found that upon increasing the concentration of insulin and glucose in the medium of VAT explants, the expression of omentin-1 is significantly decreased. This finding is in line with several studies showing that the reduced omentin-1 in adipose tissue may contribute to the development of insulin resistance and T2DM. However, it should be noted that our findings relate only to obese patients with MASH without T2DM. Therefore, it would be of interest if we also determine the levels of omentin-1 in VAT of diabetic or lean patients. Additionally, one of the limitations of our study pertains to the regulation of omentin-1 in response to glucose and insulin actions in the context of MASH. Therefore, the physiologic and pathologic significance of our findings remain to be elucidated.

## Conclusions

Obesity is a significant risk factor for MASLD, where the expansion of visceral adipose tissue contributes to numerous pathological events, including the dysregulation of adipocytokines. The present study provides evidence that reduced omentin-1 level is associated with obesity-related MASLD. Although the cause-and-effect relationship is still unclear, we are still able to show that omentin-1 is an adipocytokine that plays a significant role in the VAT-liver crosstalk. As an endocrine factor, we report herein that VAT omentin-1 has a protective role against fat-laden hepatocytes showing inflammation, ER stress, and oxidative stress. Locally, omentin-1 was able to regulate obese VAT mechanisms, especially insulin-glucose impairment. These beneficial effects of omentin-1 might be attributed to suppression of MAPK signaling and the inhibition of NF-κB activation. Further studies are required to elucidate the biological activity of omentin-1 in obesity-related MASLD with a focus on specific receptor identification, which could then eventually facilitate new drug development.

### Supplementary Information

**Additional file 1.** A mean difference (MD) the scatter plot displays log 2 fold change versus log 2 expression using limma. Upon setting the threshold for the DEGs at corrected p-value < 0.05 and log 2 fold change (FC) of |1|, 110 upregulated and 35 downregulated genes were identified.

**Additional file 2.** Summary of the simple in silico protein discovery strategy used in the study. (**a**) Layout of the in silico funnel strategy and the criteria used. (**b**) Venn diagrams illustrating the different datasets used to identify candidates satisfying our selection criteria. Abbreviations: MO, morbidly obese.

**Additional file 3.** Clinical and laboratory characteristics of the study groups.

## Data Availability

The dataset GSE58979 used in this study is available in the GEO repository.

## Abbreviations

ALT: Alanine aminotransferase
AST: Aspartate aminotransferase
AT: Adipose tissue
AUF: Arbitrary unit of fluorescence
BCA: Bicinchoninic assay
BiP: Binding of immunoglobulin protein
BMI: Body mass index
CD: Control diet
*CHOP*: CCAAT/enhancer-binding protein homologous protein
ER: Endoplasmic reticulum
ERK: Extracellular signal regulated kinase
FFA: Free fatty acids
GGT: Gamma-glutamyl transferase
GSH: Reduced glutathione
GSSG: Oxidized glutathione
HDL: High-density cholesterol
HFD: High-fat diet
*HPRT*: Hypoxanthine–guanine phosphoribosyltransferase
JNK: c-Jun N-terminal protein kinase
MAPK: Mitogen-activated protein kinases
MASLD: Metabolic dysfunction-associated steatotic liver disease
MASH: Metabolic-associated steatohepatitis
MO: Morbidly obese
NF-κB: Nuclear factor kappa B
NAFLD: Nonalcoholic fatty liver disease
SAT: Subcutaneous adipose tissue
SOD: Superoxide dismutase
TNF-α: Tumor necrosis factor alpha
T2DM: Type 2 diabetes mellitus
VAT: Visceral adipose tissue
