# Supplementary figures and images for: The potential role of omentin-1 in obesity-related metabolic dysfunction-associated steatotic liver disease: evidence from translational studies
Source: J Transl Med. 2023 Dec 11;21:906. doi: 10.1186/s12967-023-04770-8 (PMC10714452; doi:10.1186/s12967-023-04770-8)

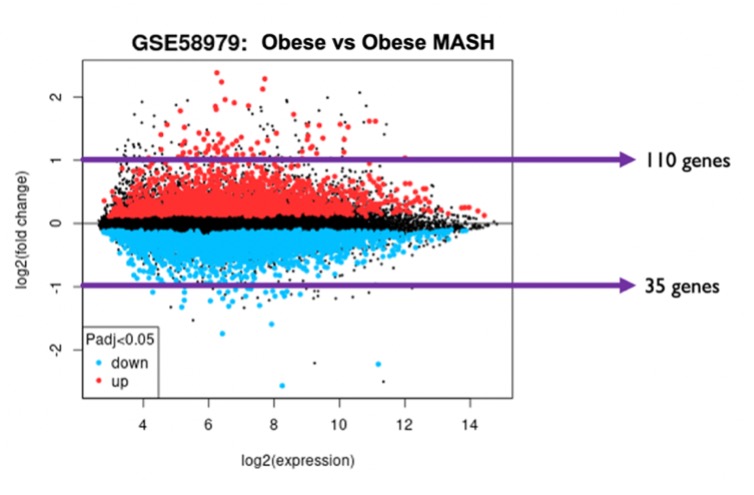

Supplement: Supplementary file 1 — Additional file 1. A mean difference (MD) the scatter plot displays log 2 fold change versus log 2 expression using limma. Upon setting the threshold for the DEGs at corrected p-value < 0.05 and log 2 fold change (FC) of |1|, 110 upregulated and 35 downregulated genes were identified. [file 12967_2023_4770_MOESM1_ESM.jpg]

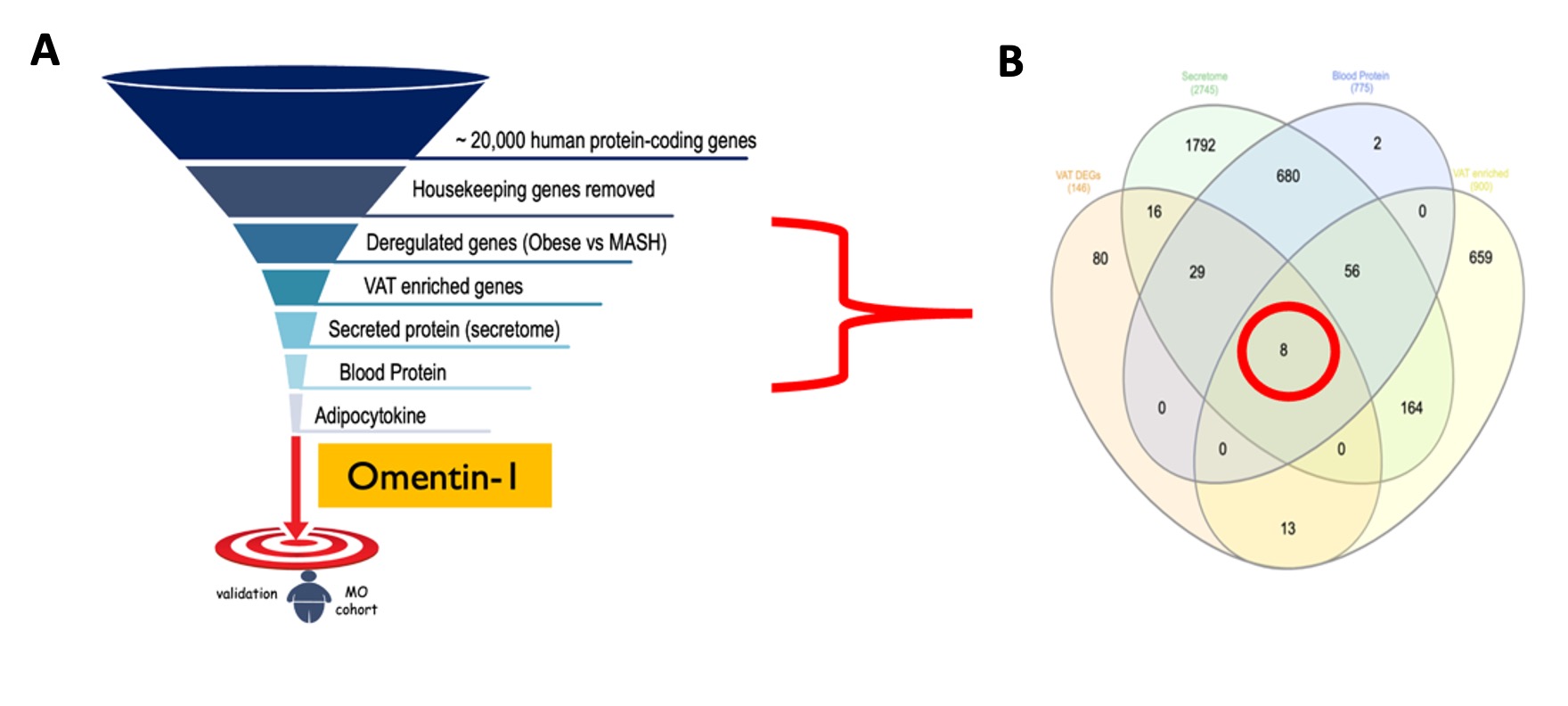

Supplement: Supplementary file 2 — Additional file 2. Summary of the simple in silico protein discovery strategy used in the study. (a) Layout of the in silico funnel strategy and the criteria used. (b) Venn diagrams illustrating the different datasets used to identify candidates satisfying our selection criteria. Abbreviations: MO, morbidly obese. [file 12967_2023_4770_MOESM2_ESM.jpg]
