# Supplementary material for: The potential role of omentin-1 in obesity-related metabolic dysfunction-associated steatotic liver disease: evidence from translational studies
Source: J Transl Med. 2023 Dec 11;21:906. doi: 10.1186/s12967-023-04770-8 (PMC10714452; doi:10.1186/s12967-023-04770-8)
Supplement: Supplementary file 3 — Additional file 3. Clinical and laboratory characteristics of the study groups. [file 12967_2023_4770_MOESM3_ESM.docx]

**Additional file 3. Clinical and laboratory characteristics of the study groups.**

| **Variable** | **Obese**  **(Ob)**  **(n = 19)** | **Obese MASH (Ob-M)**  **(n = 20)** | **Obese MASH with Fibrosis**  **(Ob-MF)**  **(n = 16)** | ***p* value** |
| --- | --- | --- | --- | --- |
| Age (years) | 39.3 ± 8.65 | 46.8 ± 11.9 | 44.9 ± 11.3 | 0.08 |
| Sex (female) | 19 (100%) | 16 (80%) | 9 (56%) | 0.006 |
| BMI (kg/m^2^) | 41.0 ± 5.6 | 43.3 ± 4.9 | 46.4 ± 8.4 | 0.09 |
| Fasting glucose (mg/dL) | 99.5 ± 10.6 | 118.5 ± 37.0* | 114.1 ± 25.6^#^ | 0.04 |
| T2DM (yes) | 3 (16%) | 6 (30%) | 4 (25%) | 0.387 |
| AST (U/L) | 18.6 ± 5.12^#^ | 25.4 ± 10.5 | 33.3 ± 20.5^#^ | 0.02 |
| ALT (U/L) | 18.1 ± 8.7 | 35.2 ± 19.3** | 44.7 ± 37.9^##^ | <0.001 |
| GGT (U/L) | 21.0 ± 13.6 | 36.3 ± 24.0 | 45.9 ± 36.1^##^ | 0.005 |
| ALP (U/L) | 76.53 ± 34.4 | 78.4 ± 19.4 | 85.3 ± 19.8 | 0.15 |
| Albumin (g/dL) | 3.91 ± 0.76 | 4.16 ± 0.28 | 4.29 ± 0.38 | 0.08 |
| Platelets (X10^9^/L) | 256.8 ± 92.7 | 284.7 ± 66.7 | 226.1 ± 44.6^∞^ | 0.03 |
| Total Cholesterol (mg/dL) | 174.2± 31.2 | 208.0 ± 37.9* | 207.1 ± 49.0^#^ | 0.03 |
| HDL cholesterol(mg/dL) | 59.5 ± 50.9 | 45.6 ± 11.4 | 45.7 ± 12.0 | 0.26 |
| Triglycerides (mg/dL) | 105.2 ± 34.9 | 147.2 ± 58.4* | 150.0 ± 74.3^#^ | 0.02 |
| AST/ALT Ratio | 1.12 ± 0.44 | 0.80 ± 0.21* | 0.82 ± 0.20^#^ | <0.01 |
| Iron (mcg/dL) | 66.6 ± 34.1 | 73.5 ± 31.9 | 66.5 ± 20.0 | 0.871 |
| Transferrin (mg/dL) | 260.0 ± 71.2 | 280.7 ± 44.0 | 306.9 ± 50.13^#^ | 0.05 |
| Ferritin (ng/mL) | 37.8 ± 23.6 | 69.6 ± 84.9 | 72.5 ± 62.3 | 0.39 |

Data are shown as mean ± SD for continuous variables, number (%) for binary variables, and frequency for categorical variables. ANOVA was used to test for significant differences within continuous variables that were normally distributed while Kruskal-Wallis with Dunn post-test when not normally distributed. Chi-Square test was used for categorical variables. **p < 0.001 *vs* Ob-M, *p < 0.05 *vs* Ob-M, ^##^p < 0.001 *vs* Ob-MF, ^#^p < 0.05 *vs* Ob-MF, ^∞^p < 0.05 Ob-M *vs* Ob-MF. Abbreviations: BMI, body mass index; ALT, alanine aminotransferase; AST, aspartate aminotransferase; GGT, gamma-glutamyl transferase; T2DM, type 2 diabetes mellitus; HDL, high-density cholesterol.
